# Progress in Pathogenesis of Immunoglobin A Nephropathy

**DOI:** 10.7759/cureus.8789

**Published:** 2020-06-23

**Authors:** Jemima C Stanley, Hong Deng

**Affiliations:** 1 Pathology, Zhejiang University School of Medicine, Hangzhou, CHN

**Keywords:** autoimmune, gut-kidney, iga1, iga nephropathy, treatment

## Abstract

The understanding of the pathogenesis of any disease is the key to effective and specific treatment of the disease. immunoglobulin A (IgA) nephropathy is an autoimmune disease of the kidney. Oxford MEST classification is commonly used to stratify patients according to the severity of the disease. Patients with IgA nephropathy seem to produce anti-GalNAc antibodies against a particularly defective IgA1. This immune complex deposits in the kidneys, leading to a type 3 hypersensitivity reaction which ultimately damages the kidneys. People of a certain genetic background and who experience upregulation of certain defective receptors seem to develop primary IgA nephropathy. Secondary IgA nephropathy could be due to dysbiosis of the microbiota in the gut, compromised gut immunity or other gut pathologies, pulmonary function abnormalities, or amyloidosis. Overproduction of IgA due to plasma cell dyscrasia or reduced clearance of IgA due to liver abnormalities could also be potential causes. Genes that predispose individuals to IgA nephropathy and intestinal abnormalities, such as Celiac disease, seem to overlap and these people tend to have a poorer prognosis and need to be placed on more intensive treatment regimens. IgA Vasculitis seems to be a systemic form of IgA nephropathy, whereby IgA deposits systemically and leads to multiple disease manifestations. Patients in high-risk groups could also be prophylactically screened for the disease and closely monitored by immunohistochemical methods such as an enzyme-linked immunosorbent assay (ELISA) or identified by genetic testing. Currently, the major treatment regimens involve supportive therapy or immunosuppressive therapy which has major side effects. More specific treatment methods such as monoclonal antibodies, immunoglobulin replacement therapy, or low-antigen-content diet could also be looked into as potential treatment options. Stem cell replacement, by way of bone marrow transplant and tonsillectomy, has been suggested as a treatment option in patients with indications.

## Introduction and background

Immunoglobin A nephropathy (IgAN), also known as Berger’s disease, is an autoimmune disease characterized by IgA deposits in the kidney which leads to inflammation and damage of the kidney. Generally, IgA nephropathy is the most common type of primary glomerulonephritis [1]. It can lead eventually to kidney failure if not treated immediately. Urinary abnormalities indicating an underlying renal pathology elicit concern and make the patient seek help. Urine analysis would probably reveal macrohematuria or proteinuria which would have manifested clinically as acute nephritic syndrome or edema commonly due to nephrotic syndrome, respectively. Renal biopsy is necessary to make the definitive diagnosis, after which classification of the severity of the disease should be made in order to ascertain how to treat the patient. Classification is based on the percentage of the glomeruli with pathological variable, proteinuria (g/day), and histological grading in Japan [2]. According to the Oxford MEST classification which was published in 2009 and is more commonly used clinically, mesangial hypercellularity, segmental glomerulosclerosis, endocapillary hypercellularity, and tubular atrophy/interstital fibrosis have to be considered for classification [3]. Treatment regimens, prognosis, and the rate of kidney deterioration can be determined based on this. Prophylaxis would be the best option, especially in populations prone to IgA nephropathy, as any specific treatment or cure is currently unavailable. Supportive therapy and immunosuppressive therapy is commonly used in the management of this disease. People who receive transplants are also likely to develop IgA nephropathy due to IgA deposition in the donor kidney [4]. Further studies on pathogenesis and research based on that pathogenesis are required to overcome the issue of lack of specific treatments or proper prophylactic screening methods for the disease. 

## Review

Genetic basis

The reported prevalence of IgA nephropathy is higher in Asian populations than in Caucasians and generally has the highest incidence in the second and third decades of life. According to an epidemiological study in China on 13,519 renal biopsies, IgAN accounted for 33.19% of total renal biopsy diagnoses and 45.26% of primary glomerular diseases. In contrast, in the United States and western Europe, IgAN accounts for 10% of total renal biopsy diagnoses and 30% of primary glomerular diseases [5]. It follows an autosomal dominant inheritance with incomplete penetrance, indicating that it is quite obviously an inherited disease and involves familial clustering. Serum levels of circulating IgA 1 with O-glycosylated hinge regions are elevated in individuals prone to develop IgAN [6].

General pathogenesis

The pathogenesis of the disease is key to understanding how to prevent and treat it. The general pathogenesis of IgA nephropathy, whether primary or secondary, is due to the increased deposition of IgA in the kidney, leading to kidney dysfunction. The negative charge of the mesangial basement membrane triggers the deposition of positively charged immunoglobulins along the mesangial matrix [7]. A particularly defective IgA1 molecule with an O-glycosylated hinge region (GdIgA1) acts as an antigen for self-reactive IgA or IgG antibodies [8]. This forms an immune complex that deposits at the mesangium and leads to activation of the complement system by C3 after which a type 3 hypersensitivity reaction occurs. If self-reactive IgG antibodies attach to the hinge region, the prognosis of the disease becomes worse [9]. The hypersensitivity reaction leads to the destruction of the mesangium, causing proteinuria, hematuria, and eventual kidney failure. IgG co-deposition leads to lower creatinine reabsorption rates and lower rates of remission.

There also seems to be reduced clearance of IgA, especially when there are concurrent hepatic pathologies as the liver clears the IgA from the circulation. IgA is also cleared by CD89 (FcRI) mediated endocytosis and catabolism by myeloid cells. Not only is CD89 downregulated in IgA nephropathy, there also seems to be a lesser affinity for it, leading to lesser clearance and therefore, deposition in the kidney [10].

**Figure 1:**
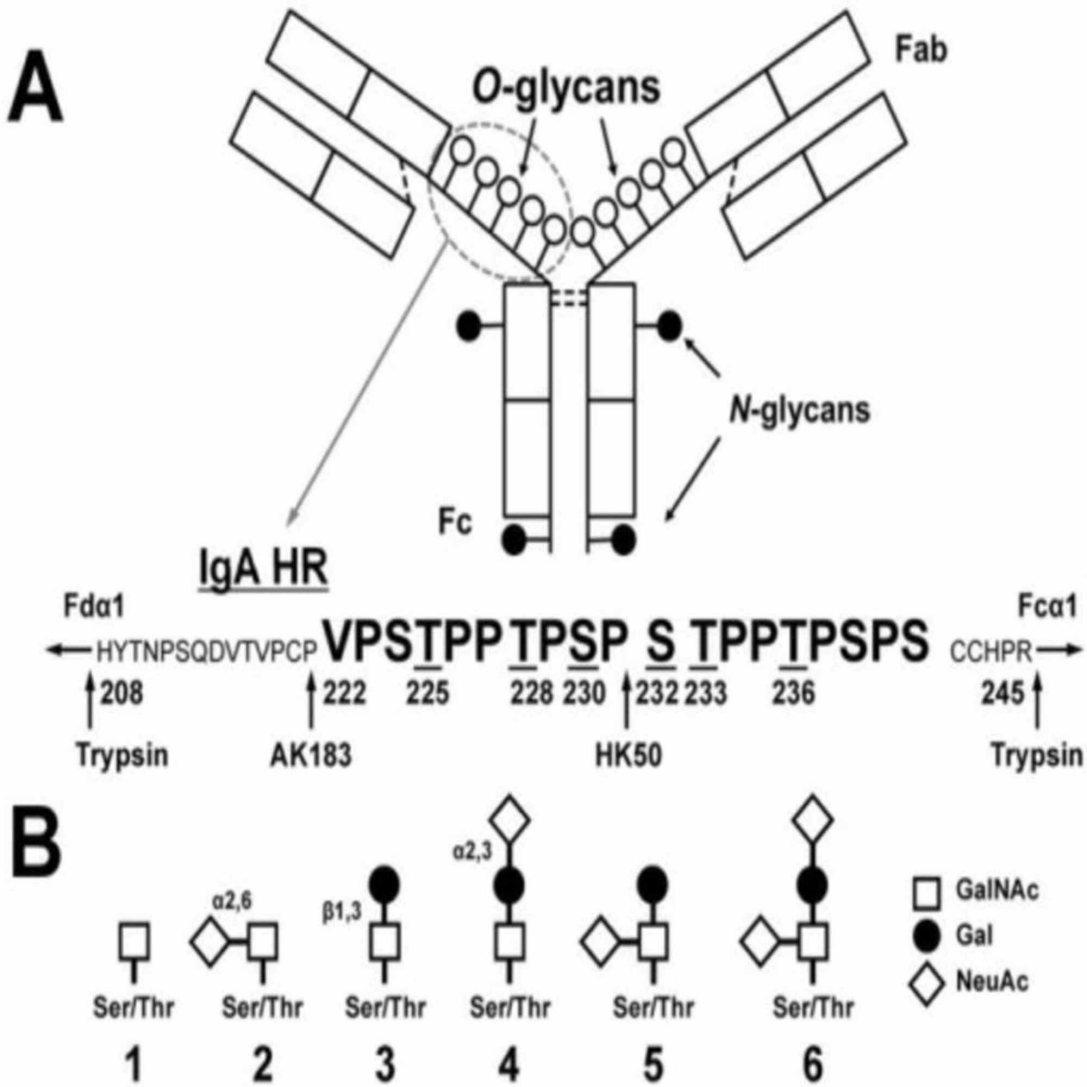
Structure of immunoglobulin Figure adapted from Novak et al. [11]

There are asymptomatic patients with IgA deposition in the kidneys. Then what makes some patients progress to IgA nephropathy while some patients remain healthy and asymptomatic? Anti-GalNAc autoantibodies. Patients with IgA nephropathy had elevated serum levels of IgA1 with galactose-deficient glycans (see 1 and 2 from Figure 1 below) [11]. These galactose deficient glycans have GalNAc exposed. This allows the binding of Anti-GalNAc IgA or IgG, which then subsequently leads to the deposition of immune complexes in the mesangium of the kidney, leading to hypersensitivity 3 reaction.

**Figure 2:**
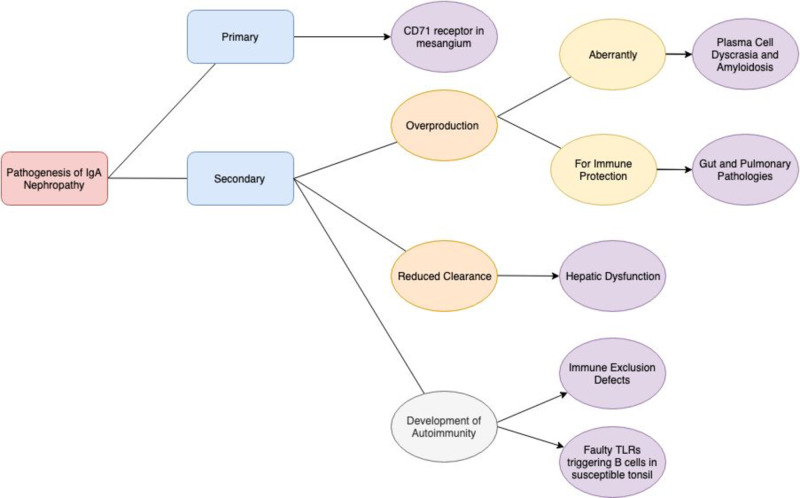
Summary figure showing the “pathogenesis of IgA nephropathy”

Autoantibodies are generally found to bind short fragments subtracted from larger glycans, but there was no recognition of the same short fragment in the context of the whole chain by the antibody [12]. The galactose deficiency thus makes the glycan shorter, increasing the chances of autoantibodies being able to bind to it. About 30% of the found antibodies recognize the core (hidden, inner part) of larger glycans. The deficiency of the galactose exposes this core GalNAc for autoantibody binding. Genes that predispose individuals to IgA nephropathy and intestinal diseases seem to overlap. A summary figure has been included below in Figure 2 in order to give a general insight into the pathogenesis of IgA nephropathy.

Primary IgA nephropathy

Primary IgA nephropathy is thought to be an autoimmune disease, with genetics and family history playing a huge part since first-degree relatives of the patients also seem to have abnormalities in IgA, even without progression to disease. There also seems to be a clustering of birthplaces, suggesting the "founder effect" [13]. There also seems to be some upregulation of aberrant IgA receptors and defects in receptors that lead to the clearance of IgA in the kidney mesangium itself. The CD71 transferrin receptor, not generally expressed in healthy glomeruli, seems to be upregulated. β-1,4-galactosyltransferase is a constitutively expressed mesangial cell IgA receptor [14]. Its catalytic domain binds to the Fc portion of IgA and clears it. Patients with IgA nephropathy seem to have an upregulation of this β-1,4-galactosyltransferase receptor, but deposition still occurs because the rate of IgA deposition has exceeded the rate of clearance due to receptor having reached saturation.

Secondary IgA nephropathy

IgA nephropathy as a secondary disease is due to a disruption in the normal production or clearance of IgA; it is produced in excess or cleared to a lesser extent. This is secondary to other diseases or pathological abnormalities and resolves when the main disease is treated.

Gut-Kidney Axis

The gut-kidney axis is an interaction that is a part of normal physiology. Uremia affects the normal flora of our intestines, and toxins that affect the kidney arise because of these microbiotas [15]. This indicates the bidirectional relationship between the gut and kidney. If there is increased intestinal permeability, toxins may enter the body and damage the kidney. Since the predominant immunoglobulin present at mucosal surfaces is IgA, it causes IgA Immune complexes to deposit in the kidney when there are disruptions in the normal gut-kidney axis. Therefore diseases like irritable bowel syndrome, Crohn’s disease, inflammatory bowel disease, Celiac disease and other such gastrointestinal tract diseases may be associated with IgA nephropathy [16].

Recurrent bacterial infections or dysbiosis of the microbiota (the normal flora of our intestines) leads to the pathogenesis of IgA nephropathy [17]. The gut flora competes with pathogenic bacteria that pass through our gut and kills them so that it does not enter our bloodstream and cause systemic infection. Dysbiosis or imbalance in these bacteria means increased intestinal permeability due to disruption of the intestinal barrier which triggers the immune system to overproduce IgA [18]. Increased intestinal permeability means increased Lipopolysaccharides (LPS) exposure which activates TLR4 in B lymphocytes causing the methylation and subsequent inactivation of core 1 beta3-Gal-T specific molecular chaperone (Cosmc). Cosmc is essential for galactosyltransferase activity. Therefore, elevated levels of galactose-deficient glycans are produced [19]. Microbiota dysbiosis leads to increased production of IgA and in particular, galactose deficient IgA. 

Overexpression of B-cell survival factor (BAFF) which is necessary for the survival of B cells, and a proliferation-inducing ligand (APRIL), which is necessary for IgA synthesis, seem to be present in both individuals with inflammatory bowel disease and IgA nephropathy [20, 21]. There is some evidence that the B-cell survival signal disturbs the normal balance of the microbiota [22]. There is a minimal increase in the serum concentration of IgA. 

Bacterial metabolites of bacteria such as short-chain fatty acids (SCFA) play a protective role in ischemia-reperfusion induced acute kidney, whereas toxic metabolites such as p-cresol sulfate and indoxyl sulfate are can cause the release of pro-inflammatory cytokines and thereby accelerate the progression of kidney injury. Once kidney damage has started to occur, progression to end-stage renal disease is really rapid. Furthermore, there is increased uric acid in the blood due to kidney dysfunction which means the colon has to adapt to secrete it. Therefore, bacterial families possessing urease, uricase, phenol, and indole-forming enzymes increase, whereas the SCFA-forming bacteria are contracted in ESRD patients [23]. Increased secretion of ammonia and urea into the gut alters the pH of the gut, making it more alkaline than it already is, causing the growth of some pH-sensitive bacteria. According to Vaziri et al., analysis of the intestinal microbial flora of 24 patients with ESRD compared with 12 healthy controls, Brachybacterium, Catenibacterium, Enterobacteriaceae, Halomonadaceae, Moraxellaceae, Nesterenkonia, Polyangiaceae, Pseudomonadaceae, and Thiothrix families showed a remarkable increase in patients with ESRD [24]. According to a recent study, low counts of Bifidobacterium and high number of Streptococceae were also observed in patients with kidney disease [25]. Enterococcus and Lactobacillus were found also at the highest levels in healthy controls and not in patients with kidney disease.

Celiac Disease: A Disease Modifier not a Secondary Disease

Celiac disease is a type of chronic mucosal inflammation of the small intestine leading to villous atrophy and therefore, malabsorption. These occur due to exposure to wheat gluten or rye and barley proteins [26]. It is also characterized as an autoimmune disease, much like IgA nephropathy. High levels of IgA against food antigens in patients with IgA nephropathy have led to the possibility of the link between the two diseases. A gluten-free diet does seem to have an improvement in kidney function, even when the patient is only diagnosed with IgA nephropathy and celiac disease is not yet confirmed [27]. Issues with mucosal antigen exclusion and systemic hypersensitivity (hyperresponsiveness) in primary IgA nephropathy make antibodies against food antigen. This triggers patients who are already susceptible to Celiac disease, which further increases IgA and increases mesangial deposition of IgA. This further exacerbates the IgA nephropathy, leading to a further decrease in kidney function at a faster rate. Therefore, Celiac disease is more of a disease modifier than an actual cause of secondary IgA nephropathy. 

Immune Exclusion Defects

Immune exclusion is when a certain level of tolerance to the antigens in the gut is acquired, even when they enter the bloodstream, due to repeated exposure and therefore desensitization over the years [28]. Secretory IgA (SIgA) facilitates this immune exclusion by neutralizing these antigens and preventing their interaction with the epithelium [29]. These immune complexes are internalized by Antigen Presenting Cells in Peyer’s patches, masking the epitopes of the antigen, in order to not trigger an overwhelming immune response. Oral tolerance develops based on antigen dosage. High doses would cause the T cells that recognize these immune complexes to undergo anergy. Low doses would stimulate the regulatory T cells. There has even been mention of suppressor cells that actively suppress the immune system when it acts against food antigens back in the 1970s [30]. The memory of these suppressor cells could be passed on through the generations. Therefore, tolerance towards both orally administered antigens and normal flora of the gut can be achieved by sIgA directed immune exclusion and immune suppression in general [31]. This establishes homeostasis of the intestinal immune system. Any disturbance to this homeostasis leads to autoimmune diseases which cause increased production of IgA and could potentially lead to IgA nephropathy.

Hepatic Dysfunction

Hepatic IgA dysfunction is said to be the most common cause of secondary IgAN, although patients may remain asymptomatic [32]. It might occur due to the fact that the damaged liver is unable to clear the IgA, IgA immune complexes, small amount of other immunoglobulins, and C3, leading to its deposition in the kidney via FcαR on Kupffer cells and asialoglycoprotein receptor on hepatocytes [33]. There is also an increase in gut permeability due to liver damage which leads to increasing serum IgA against common food and microbial antigens [34]. The damaged liver is also able to assert less control over the production of IgA [35]. 

Pulmonary-Renal Syndrome

Since the respiratory tract is also lined with mucosa, the predominant immunoglobulin protecting the respiratory tract is IgA. As such, infections involving the lung causing vasculitides, bronchiolitis, capillaritis, alveolitis, and pulmonary hemorrhage (diffuse alveolar hemorrhage), and even tuberculosis, though less common, have caused secondary IgA nephropathy [36]. In some cases, pulmonary diseases seem to resolve when they are treated with corticosteroids for the IgA nephropathy, suggesting the existence of a pulmonary-kidney axis [37]. 

IgA Vasculitis: A Systemic Form of IgA Nephropathy?

IgA vasculitis is the most common form of vasculitis in children, and commonly occurs after an upper respiratory tract infection [38]. It is an IgA-mediated systemic small-vessel vasculitis. The IgA deposition in the small vessel walls leads to symptoms involving the skin, joints, gastrointestinal tract and kidneys [39]. This disease seems to be the systemic form of IgA nephropathy. Both diseases share similar histopathology, pathogenesis and immunogenetics. They have deposition of similar IgA subtypes and complement deposition [40]. IgA nephropathy is usually a disease that is common in adults. If a child is diagnosed with IgA nephropathy, tests for HSP should also be done.

Amyloidosis and Plasma Cell Dyscrasia

During plasma cell dyscrasias there is a dysfunction of the plasma cell so it can overproduce antibodies by not responding to the stop signal or produce part of an antibody and release it. The deposition of the immunoglobulin light chain could lead to amyloidosis as these immunoglobulins tend to be glycosylated [41]. Therefore, they could cause an increase in Anti-GalNAc autoantibodies which would also cause IgA nephropathy. One previously observed case had IgG plasma cell dyscrasia with IgA nephropathy. IgG co-deposition only worsens the symptoms and prognosis of IgA nephropathy. The patient’s IgA nephropathy was cured after the treatment of the IgG plasma cell dyscrasia [42]. The other patient had urethral amyloidosis with IgA nephropathy but their IgA nephropathy was also cured after the treatment of the amyloidosis [43].

Tonsils

Toll-like receptors (TLR) help to discriminate between self and non-self antigens and trigger suitable immune mechanisms. Exogenous microbial antigens seem to activate TLR9 in the mucosa which activates the B cells in the tonsils, which in turn leads to the production of nephritogenic GdIgA1, especially after insult to the tonsils such as an infection [44].

This leads us to believe that IgA nephropathy manifests as a secondary disease in many cases and treatment of the primary disease would automatically cure the IgA nephropathy. In patients with pre-existing kidney damage or disease, the diseases have to be treated simultaneously to prevent progression to chronic kidney diseases.

Prophylaxis and treatment

Currently, there is no proper prophylactic treatment for IgA nephropathy. In the future, people who have a family history of IgA nephropathy can screen for elevated IgA 1 levels using immunohistochemical methods such as an enzyme-linked immunosorbent assay (ELISA). Previously, lectin-dependent assays were used to screen for Galactose-deficient IgA1 (Gd-IgA1) which proved to be ineffective. Now, Gd-IgA1-specific monoclonal antibody KM55 seems to be very effective in localizing Gd-IgA1 [45]. These KM55 antibodies can be used for screening in high-risk individuals and then monitoring them for early detection of disease onset. Then begin monitoring of the patients who are prone to develop the disease and treat them as quickly as possible. If there are intestinal, hepatic, or B cell pathologies in the patient, then monitor the patient for IgA nephropathy. Genetic and Epigenetic testing could also be done in children with a family history of IgA nephropathy to see if they are prone to developing the disease.

Firstly, it is important to determine whether IgA nephropathy is a primary or secondary disease. If it is a secondary disease, treatment of the main disease should resolve the IgA nephropathy. If it is a primary disease, it is treated currently by either supportive therapy or immunosuppressive therapy. Supportive therapy is administered to patients with minimal urinary abnormalities. This includes renin-angiotensin-aldosterone system inhibitors in patients with hypertension and proteinuria and regular follow-up to monitor disease progression and hypertension. In patients with rapidly progressing disease, immunosuppressive therapy is required. This could potentially lead to severe complications, however, because of the increased occurrence of infections in these patients due to their immunocompromised state. Novel therapies, such as the NEFIGAN trial, include administering budesonide directly to gut-associated lymphoid tissues (GALT) which have proven to be effective due to the gut-kidney axis [46]. 

Low-antigen-content (LAC) diet in the treatment of patients with IgA nephropathy has also shown to be effective [47]. The gut-kidney axis described above also further elicits the importance of further research into the LAC diet as a more commonly available treatment option. There seemed to be a marked reduction or disappearance of proteinuria and biopsy revealed a significant reduction in mesangial deposition of Immunoglobulins and complement C5, all predictors of poor prognosis in patients with IgA nephropathy. The results could be attributed to the reduction of nephritogenic food antigen input and the relation between the gut and kidney, which was described in detail earlier.

Although the effectiveness of tonsillectomy as a treatment for IgA nephropathy remains ambiguous, it could be explored as a treatment option. Tonsillectomy seems to improve the urinary findings and the overall renal function in some IgA nephropathy patients [48]. It does not cause any marked deficiency in immunity. Tonsillectomy could be indicated in patients whose renal function declined after tonsillitis.

More research could be put into more specific treatment regimens. Upregulation of receptors that clear IgA and IgA immune complex deposits such as β-1,4-galactosyltransferase could be looked into. Patients could also be treated with monoclonal antibodies against the Anti-GalNAc autoantibodies, or if need be then against the defective IgA1 molecules with an O-glycosylated hinge region itself. Following this, should the patient be deficient in Immunoglobulins, Immunoglobulin replacement therapy could be done whereby serum IgG and IgA are administered systemically. Physiologically, serum IgA could become secretory IgA in the mucosa, but if this is not the case, IgA could be directly administered onto the mucosal surfaces [49]. For example, if the lung mucosa is deficient then, directly to the airways through devices similar to nebulizers and inhalers. If the gut mucosa is deficient in secretory IgA, then immunoglobulins could be administered directly to GALT, much like what has been done with administering of budesonide in the NEFIGAN trial. Alternatively, stem cell replacement could also be looked into. It has already been established that patients with IgA nephropathy have elevated serum IgA levels, especially of the IgA1 subclass, which is derived from the bone marrow. Bone marrow transplant could be considered in extreme cases where patients have other indications for the procedure, so they can produce normal IgA1 without the O-glycosylated hinge region or self-reactive autoantibodies. There has been a case that has been reported of a patient with complete remission of IgA nephropathy after bone marrow transplant [50].

## Conclusions

IgA nephropathy is caused by the deposition of IgA with an O-glycosylated hinge region, which seems to be inherited genetically, in the mesangial matrix which triggers a type 3 hypersensitivity reaction. The presence of IgG or certain complement factors only seems to worsen the prognosis. There are many secondary causes of the disease of which the main ones would be pulmonary and gastrointestinal tract infections, as IgA is the predominant mucosal immunoglobulin in the lungs and gastrointestinal tract. The secondary IgA nephropathy resolves once the primary disease is treated. The aim of the article is to give insights into the various pathogenetic pathways of IgA nephropathy, in order to create pathways for research into novel specific treatments regimens and to also emphasize the importance of the development of prophylactic screening methods, especially in areas where IgA nephropathy is widespread and rampant, so as to alleviate the number of patients who progress to end-stage renal disease.

## References

[1] Zhu L, Zhang H (2015). The genetics of IgA nephropathy: an overview from china. Kidney Dis (Basel).

[2] Yuzawa Y, Yamamoto R, Takahashi K (2016). Evidence-based clinical practice guidelines for IgA nephropathy 2014. Clin Exp Nephrol.

[3] Trimarchi H, Barratt J, Cattran DC (2017). Oxford Classification of IgA nephropathy 2016: an update from the IgA Nephropathy Classification Working Group. Kidney Int.

[4] Moriyama T, Nitta K, Suzuki K (2005). Latent IgA deposition from donor kidney is the major risk factor for recurrent IgA nephropathy in renal transplantation. Clin Transplant.

[5] Cai GY, Chen XM (2009). Immunoglobulin A nephropathy in China: progress and challenges. Am J Nephrol.

[6] Takahashi K, Horynova MS, Hall SD (2014). Enzymatic sialylation of IgA1 O-glycans: implications for studies of IgA nephropathy. PLoS One.

[7] Nangaku M, Couser WG (2005). Mechanisms of immune-deposit formation and the mediation of immune renal injury. Clin Exp Nephrol.

[8] Suzuki H, Kiryluk K, Novak J (2011). The pathophysiology of IgA nephropathy. J Am Soc Nephrol.

[9] Wada Y, Ogata H, Takeshige Y (2013). Clinical significance of IgG deposition in the glomerular mesangial area in patients with IgA nephropathy. Clin Exp Nephrol.

[10] Barratt J, Feehally J (2005). IgA Nephropathy. J Am Soc Nephrol.

[11] Novak J, Rizk D, Takahashi K (2015). New insights into the pathogenesis of IgA nephropathy. Kidney Dis.

[12] Bovin N, Obukhova P, Shilova N (2012). Repertoire of human natural anti-glycan immunoglobulins. Do we have auto-antibodies?. Biochim Biophys Acta.

[13] Kiryluk K, Novak J, Gharavi AG (2013). Pathogenesis of immunoglobulin A nephropathy: recent insight from genetic studies. Annu Rev Med.

[14] Molyneux K, Wimbury D, Pawluczyk I (2017). β1,4-galactosyltransferase 1 is a novel receptor for IgA in human mesangial cells. Kidney Int.

[15] Evenepoel P, Poesen R, Meijers B ( 2017). The gut-kidney axis. Pediatr Nephrol.

[16] Terasaka T, Uchida HA, Umebayashi R The possible involvement of intestine-derived IgA1: a case of IgA nephropathy associated with Crohn's disease. BMC Nephrol.

[17] Han L, Fang X, He Y, Ruan XZ (2016). ISN Forefronts Symposium 2015: IgA nephropathy, the gut microbiota, and gut−kidney crosstalk. Kidney Int Rep.

[18] Chen YY, Chen DQ, Chen L, Liu JR, Vaziri ND, Guo Y, Zhao YY (2020). Microbiome-metabolome reveals the contribution of gut-kidney axis on kidney disease. J Transl Med.

[19] Sun Q, Zhang J, Zhou N, Liu X, Shen Y (2015). DNA methylation in Cosmc promoter region and aberrantly glycosylated IgA1 associated with pediatric IgA nephropathy. PLoS One.

[20] Zhang YM, Zhang H (2018). Insights into the role of mucosal immunity in IgA nephropathy. Clin J Am Soc Nephrol.

[21] Corica D, Romano C (2016). Renal involvement in inflammatory bowel diseases. J Crohns Colitis.

[22] McCarthy DD, Kujawa J, Wilson C (2012). Mice overexpressing BAFF develop a commensal flora-dependent, IgA-associated nephropathy. J Clin Invest.

[23] Wing MR, Patel SS, Ramezani A, Raj DS (2016). Gut microbiome in chronic kidney disease. Exp Physiol.

[24] De Angelis M, Montemurno E, Piccolo M (2014). Microbiota and metabolome associated with immunoglobulin A nephropathy (IgAN). PLoS One.

[25] Coppo R (2018). The gut-kidney axis in IgA nephropathy: role of microbiota and diet on genetic predisposition. Pediatr Nephrol.

[26] Balakireva AV, Zamyatnin AA (2016). Properties of gluten intolerance: gluten structure, evolution, pathogenicity and detoxification capabilities. Nutrients.

[27] La Villa G, Pantaleo P, Tarquini R (2003). Multiple immune disorders in unrecognized celiac disease: a case report. World J Gastroenterol.

[28] Stokes CR, Soothill JF, Turner MW (1975). Immune exclusion is a function of IgA. Nature.

[29] Corthësy B (2009). Secretory immunoglobulin A: well beyond immune exclusion at mucosal surfaces. Immunopharmacology and immunotoxicology. Immunopharmacol Immunotoxicol.

[30] Faria AM, Weiner HL (2005). Oral tolerance. Immunol Rev.

[31] Mesin L, Sollid LM, Di Niro R (2012;3). The intestinal B-cell response in celiac disease. Front Immunol.

[32] Pouria S, Feehally J (1999). Glomerular IgA deposition in liver disease. Nephrol Dial Transplant.

[33] Hommos MS, El-Zoghby ZM (2017). Renal outcomes in patients with IgA nephropathy undergoing liver transplant: a retrospective cohort study. Transplant Direct.

[34] Pouria S, Barratt J ( 2008). Secondary IgA nephropathy. Semin Nephrol.

[35] Alghamdi SA, Saadah OI, Almatury N, Al-Maghrabi J (2012). Hepatic-associated immunoglobulin-A nephropathy in a child with liver cirrhosis and portal hypertension. Saudi J Gastroenterol.

[36] Oluwole K, Esuzor L, Adebiyi O (2012). Pulmonary hemorrhage with hematuria: do not forget IgA nephropathy. Clin Kidney J.

[37] Fortuna F, Marson B, Maciel A (2013). Pulmonary alveolitis in IgA nephropathy. Chest.

[38] Hwang HH, Lim IS, Choi BS, Yi DY (2018). Analysis of seasonal tendencies in pediatric Henoch-Schönlein purpura and comparison with outbreak of infectious diseases. Medicine (Baltimore).

[39] Yang HR (2018). What we know about Henoch-Schönlein purpura in children up to date?. J Korean Med Sci.

[40] Waldo FB (1988). Is Henoch-Schönlein purpura the systemic form of IgA nephropathy?. Am J Kidney Dis.

[41] Omtvedt LA, Bailey D, Renouf DV (2000). Glycosylation of immunoglobulin light chains associated with amyloidosis. Amyloid.

[42] Chandra A, Kaul A, Aggarwal V (2017). Immunoglobulin A nephropathy in a patient with IgG kappa light-chain myeloma. Saudi J Kidney Dis Transpl.

[43] Ojile N, Brake M (2017). Urethral amyloidosis in a patient with IgA nephropathy after renal transplant. Cureus.

[44] Suzuki Y, Suzuki H, Nakata J, Sato D, Kajiyama T, Watanabe T, Tomino Y (2011). Pathological role of tonsillar B Cells in IgA nephropathy. Clin Dev Immunol.

[45] Yasutake J, Suzuki Y, Suzuki H (2015). Novel lectin-independent approach to detect galactose-deficient IgA1 in IgA nephropathy. Nephrol Dial Transplant.

[46] Fellström BC, Barratt J, Cook H (2017). Targeted-release budesonide versus placebo in patients with IgA nephropathy (NEFIGAN): a double-blind, randomised, placebo-controlled phase 2b trial. Lancet.

[47] Ferri C, Puccini R, Longombardo G (1993). Low-antigen-content diet in the treatment of patients with IgA nephropathy. Nephrol Dial Transplant.

[48] Xie Y, Chen X, Nishi S, Narita I, Gejyo F (2004). Relationship between tonsils and IgA nephropathy as well as indications of tonsillectomy. Kidney Int.

[49] Baumann U, Miescher S, Vonarburg C (2014). Immunoglobulin replacement therapy in antibody deficiency syndromes: are we really doing enough?. Clin Exp Immunol.

[50] Park EK, Jeon JS, Noh HJ, Won JH, Park HS (2008). Complete remission of IgA nephropathy after bone marrow transplantation for acute myeloid leukaemia. NDT Plus.

